# Nutritional Education and Promotion of Healthy Eating Behaviors Among Mexican Children Through Video Games: Design and Pilot Test of FoodRateMaster

**DOI:** 10.2196/16431

**Published:** 2020-04-13

**Authors:** Ismael Edrein Espinosa-Curiel, Edgar Efrén Pozas-Bogarin, Jorge Luis Lozano-Salas, Juan Martínez-Miranda, Edwin Emeth Delgado-Pérez, Lizeth Stefania Estrada-Zamarron

**Affiliations:** 1 Centro de Investigación Científica y de Educación Superior de Ensenada Unidad de Transferencia Tecnológica Tepic Tepic, Nayarit Mexico; 2 Consejo Nacional de Ciencia y Tecnología Centro de Investigación Científica y de Educación Superior de Ensenada Unidad de Transferencia Tecnológica Tepic Tepic, Nayarit Mexico; 3 Centro de Estudios e Investigaciones en Comportamiento Universidad de Guadalajara Guadalajara, Jalisco Mexico; 4 Facultad de Salud Integral Universidad Autónoma de Nayarit Tepic, Nayarit Mexico

**Keywords:** childhood obesity, serious game, game design, nutritional education, dietary intake, healthy eating behaviors

## Abstract

**Background:**

Childhood obesity has risen dramatically in recent decades, reaching epidemic levels. Children need guidance on and support for maintaining a healthy diet and physical activity to ensure that they grow appropriately and develop healthy eating habits. Serious video games have shown positive effects on promoting the nutritional knowledge, and eating attitudes and behaviors of children; however, research about the usefulness of such games with younger children (8-10 years old) is sparse.

**Objective:**

The objective of this study was to design and test the serious video game FoodRateMaster targeting children between 8 and 10 years old. The game includes nutritional information and behavior change techniques to help children improve their knowledge of healthy and unhealthy foods, increase their intake of healthy food, and reduce their intake of ultraprocessed food. In addition, FoodRateMaster was designed as an active game to promote physical activity.

**Methods:**

An interdisciplinary team developed FoodRateMaster following an iterative methodology based on a user-centered design. A total of 60 participants (mean age 9 years, SD 0.8; 53% male) completed 12 individual gaming sessions in 6 weeks. A food knowledge questionnaire and a food frequency questionnaire were completed before and after game play. In addition, 39 of the participants’ parents answered a parent perception questionnaire after the game play.

**Results:**

Participants showed increased food knowledge from pregame (mean 56.9, SD 10.7) to postgame play (mean 67.8, SD 10.7; *P*<.001). In addition, there was a greater self-reported frequency in the consumption of cauliflower and broccoli (*P*<.001) and corn quesadillas (*P*<.001). They also indicated a lower self-reported intake of 10 unhealthy foods, including french fries (*P*=.003), candy and chocolate (*P*<.001), sweet soft cakes (*P*=.009), and soft drinks (*P*=.03). Moreover, most of the parents who answered the parent perception questionnaire agreed that their children showed greater interest in explaining why they should avoid some unhealthy foods (67%, 26/39), in distinguishing between healthy and unhealthy foods (64%, 25/39), and in the intake of fruits (64%, 25/39) and vegetables (59%, 23/39). Finally, 14 parents stated that they introduced some changes in their children's diet based on the comments and suggestions they received from their children.

**Conclusions:**

In an initial evaluation, children between 8 and 10 years old indicated an increased level in nutritional knowledge and their self-reported frequency intake of two healthy foods, and a decreased level in their self-reported intake of 10 unhealthy foods after playing FoodRateMaster. Moreover, the participants’ parents agreed that FoodRateMaster positively influenced their children’s attitudes toward several healthy eating behaviors. These results support that health games such as FoodRateMaster are viable tools to help young children increase their food knowledge and improve dietary behaviors. A follow-up randomized controlled trial will be conducted to assess the medium- and long-term effects of FoodRateMaster.

## Introduction

### Background

The incidence of childhood obesity has risen dramatically in recent decades, reaching epidemic levels [1,2]. In Mexico, 35.6% of school-aged children are overweight or obese [3]. Obesity in childhood can lead to a variety of clinical disorders with potentially severe consequences for emotional and physical health [4,5]. To reduce childhood obesity, it is recommended to increase the intake of healthy food (eg, fruits and vegetables) and reduce the intake of unhealthy food (eg, chips and soft drinks) [6]; however, many children fail to meet these recommendations. For example, only 43.5% of Mexican children meet the recommended intake of fruits, and 22% meet the recommended intake of vegetables [7]. In addition, almost 60% of Mexican children consume an excessive quantity of added sugars, and 79% exceed the recommended intake of saturated fat [8].

Children need guidance on and support for maintaining a healthy diet and physical activity to ensure that they grow appropriately and develop healthy eating behaviors [6]. Serious games are an emerging complementary intervention strategy to fulfill that need by providing exciting, innovative, and enticing methods for attracting attention, educating, and promoting changes in attitudes and human behaviors [9].

Serious video games can have positive effects on the nutritional knowledge, eating attitudes and behaviors, dietary intake, and physical activity of children [9,10]. For example, Johnson-Glenberg and Hekler [11] assessed the effects of the video game Alien Health among children aged 10 to 11 years. They identified that players of the game increased their nutrition knowledge and their knowledge regarding the US Department of Agriculture MyPlate guidelines [12]. A subsequent study showed that playing the video game Alien Health for an hour also resulted in better knowledge of the five most important macronutrients of foods among children aged 10-13 years [13]. Holzmann et al [14] demonstrated that 12 to 14-year-old children improved their nutritional and physical activity knowledge after playing the video game Fit, Food, Fun. Marchetti et al [15] assessed the effects of the video game Gustavo in Gnam’s Planet among children aged 14 to 18 years, indicating that players increased their nutrition knowledge and intake of white meat, eggs, and legumes, and decreased their intake of sugar-containing packaged snacks.

Serious video games might also be an appropriate educational tool to change children’s attitudes about food. Schneider et al [16] showed that students aged between 8 and 12 years playing the video game Fitter Critters for 1 week showed a significant increase in positive attitudes toward healthy eating and self-efficacy in making healthy food choices. Some studies have also explicitly examined whether playing serious video games changes children’s eating behaviors and food intake. Baranowski et al [17] found that children aged 10-12 years who played the games Escape from Diab and Nanoswarm: Invasion from Inner Space increased their intake of fruits and vegetables. Another study showed that playing the video game Creature 101 resulted in significant decreases in the frequency and amount of consumption of sugar-sweetened beverages and processed snacks among children aged between 11 and 13 years [18]. Moreover, Sharma et al [19] found that subjects aged 9-11 years playing Quest to Lava Mountain for 6 weeks decreased their sugar consumption and increased their nutrition/physical activity attitudes. In addition, other studies have reported the design phase of video games such as Aquamorra [20] and Pickit! and Cookit! [21], or have described initial efficiency evaluation results for games such as the Space Adventure Game [22].

To successfully achieve knowledge improvement and behavior change in children, serious video games usually combine behavior change techniques such as motivational messages, personal goals, problem-solving, and self-control activities with gamification elements that try to improve engagement and “fun”, such as stories, rewards, feedback, levels, and challenges [9].

Although positive results were obtained by the studies mentioned above, there is still a need to understand the application and limitations of such games as well as how to improve their effectiveness in consideration of the characteristics, capacity, and interests of children [9]. In particular, we identified that there is sparse evidence of the usefulness of nutrition health video games for children between 8 and 10 years old, even though it is during these years when the rates of overweight or obesity in children increase considerably [3]. Most of the video games mentioned above were evaluated with children aged 10 years or over, and the evaluations that included children between 8 and 10 years old also included older children, despite the differences in their cognitive and emotional development [23,24]. The late latency stage of children’s development can be relevant to the design of serious video games because this is the stage when children start to show more independence from their parents and develop an intense interest in rules, behavioral standards, and learning new skills. In addition, children’s self-control becomes far more reliable during this time, and they have an increased ability to remember, pay attention, think, reason, and concentrate [23,24].

### Objective

The objective of this study was to design and test the serious video game FoodRateMaster with children between 8 and 10 years old. FoodRateMaster focuses on teaching children the nutritional differences between healthy and unhealthy food and the recommended ranges that can help them to determine if they should reduce or maintain the consumption of certain foods. To our knowledge, this study is the first to design and test a game for health that encourages children aged 8 to 10 years to interact with and apply these nutritional concepts. 

The following hypotheses were established based on the notions that young children often lack an understanding of how to identify healthy and unhealthy food, and that exposure to a health video game will improve this knowledge and consequently their eating behaviors. We further assessed whether parents noted changes in their children’s attitudes toward healthy eating behaviors and implemented any dietary changes as a result.

- Hypothesis 1: Participants will be able to correctly identify a greater number of healthy and unhealthy foods.
- Hypothesis 2: Participants will self-report an increased frequency in their intake of healthy food.
- Hypothesis 3: Participants will self-report a decreased frequency in their intake of unhealthy food.
- Hypothesis 4: Participants’ parents will perceive improvements in their children’s attitudes toward healthy eating habits.

Findings from this study will have important implications for young children, who will be provided with a greater understanding of and confidence in how to identify healthy or unhealthy food, which can lead to better overall health outcomes. This study thus offers practical solutions for health practitioners and educators who design programs to teach nutrition to young children.

## Methods

### Game Design and Development

**Figure 1:**
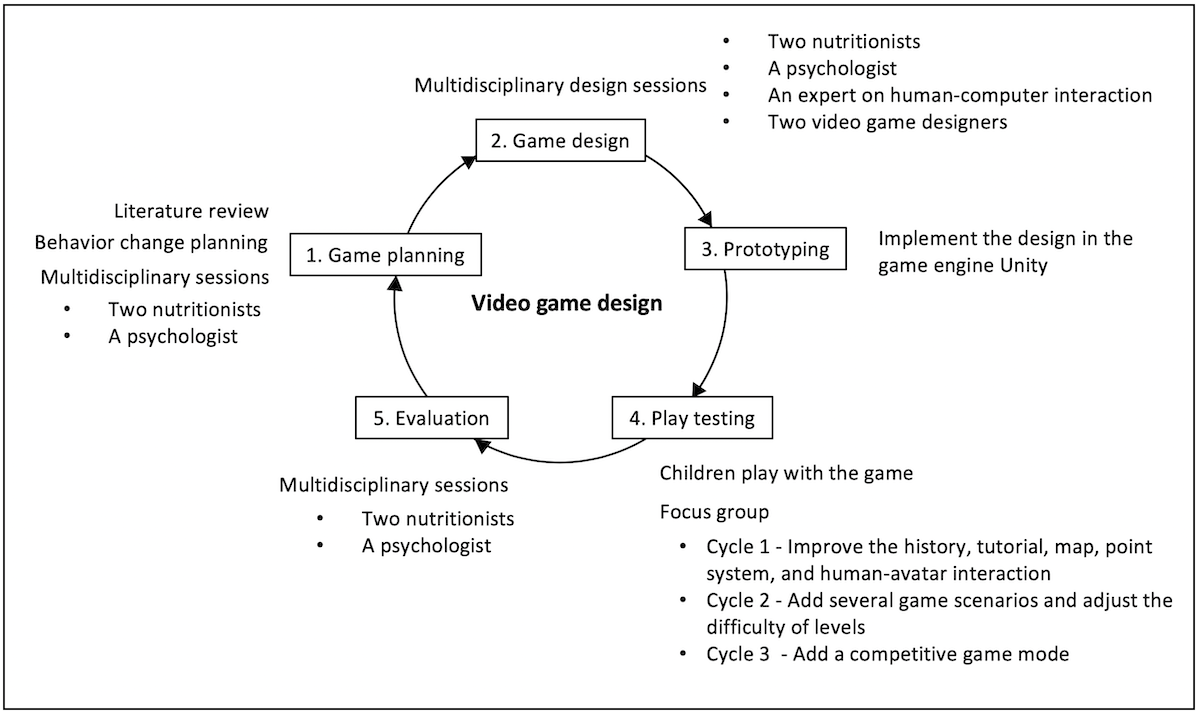
Process of designing and implementing FoodRateMaster.

To design FoodRateMaster, we used an iterative game design approach based on user-centered design methodology [25]. The methodology used has the following five steps (see Figure 1): (1) learning and behavior change planning, (2) game design, (3) prototype development, (4) play testing, and (5) evaluation. We conducted three design cycles before obtaining the version of FoodRateMaster evaluated in this study.

In Step 1, we conducted a literature review to position FoodRateMaster in the specialized serious game literature and nutrition knowledge. Additionally, we conducted several multidisciplinary design sessions with two nutritionists and a psychologist. These sessions aimed to establish or adjust the learning objectives, target behaviors, behavior change objectives, and behavior change techniques (BCTs) that could be integrated into the gameplay elements to support the behavior change objectives.

In Step 2, we conducted multidisciplinary design sessions with two nutritionists, one psychologist, one expert on human-computer interaction, and two video game designers. These sessions were conducted to propose design ideas along with game rules and mechanisms, and to define how to include the nutritional concepts and implement the selected BCTs into the gameplay elements. Based on these activities, we designed high-fidelity prototypes. We conducted 4, 2, and 2 multidisciplinary design sessions in cycles 1, 2, and 3, respectively.

In Step 3, we implemented the high-fidelity prototype based on the game design obtained in the previous step in the video game engine Unity [26].

In Step 4, children played with the prototype. They subsequently participated in a focus group where they were encouraged to talk about their game experience (eg, instructions, activities, challenges, game flow, human-computer interaction, and engaging gameplay) and propose new game elements or features. Some suggestions from cycle 1 were to improve the history, tutorial, map, point system, and human-avatar interaction. The suggestions for improving and changing cycles 2 and 3 were to add a competitive game mode, add several game scenarios, and adjust the difficulty of levels. In cycles 1, 2, and 3, there were 5, 38, and 38 children who participated, respectively. The age range of all participants was 8 to 10 years. Different children participated in each cycle, and none of these children participated in the pilot test. The play duration was 10, 15, and 15 minutes for cycles 1, 2, and 3, respectively.

Finally, in Step 5, we conducted a multidisciplinary session with the same participants as in the Step 2 sessions to discuss and analyze the obtained results, the changes suggested for the game, and the new requirements obtained in the previous step. Based on this information, we elaborated a set of recommendations to improve usability, enjoyment, player experience, game mechanics, game elements, and learning and behavior change strategies.

From the design activities described above, we identified the following requirements for FoodRateMaster: (1) mimic popular scenarios where children obtain food and include popular Mexican food; (2) focus on helping children learn how to differentiate unhealthy from healthy food; (3) mimic traditional reward mechanisms and role models; (4) encourage healthy behaviors related to the understanding of nutrition information; (5) encourage physical activity; and (6) be engaging and easy to use. 

### Description of FoodRateMaster

#### Overview of the Game

FoodRateMaster focuses on helping players understand the differences in the nutritional properties of healthy and unhealthy food, as well as the recommended ranges for food nutrients that can help them determine if they should reduce or maintain the intake of certain foods. The targeted healthy eating behaviors of FoodRateMaster are an increase in the intake of healthy food (eg, fruits and vegetables) and a reduction in the intake of ultraprocessed food (eg, snacks, sweets, soft drinks, and high-fat foods). FoodRateMaster was designed as an active game to make it more fun, improve user experience, and promote physical activity [27]. Active games require physical activity beyond that required by conventional handheld games and rely on technology that tracks body movements or reactions to progress in the game [28]. The players of FoodRateMaster need to perform basic physical movements such as squats, jumps, lateral body movements, and arm movements to avoid obstacles and classify food. To follow the body movements of the players, FoodRateMaster uses the Microsoft Kinect V2 sensor [29].

#### FoodRateMaster Mechanics and Features

The adventure of FoodRateMaster unfolds in a city where a secret agent has a mission to sabotage the plan of some evil chefs that took control of the city by giving delicious unhealthy food to children. The secret agent (an avatar controlled by the player’s movements) has to visit 6 scenarios to review the menu provided by the chefs and determine if there is a need to maintain or reduce the intake of the foods offered. The food database of FoodRateMaster includes 259 items, 179 of which are classified as healthy (eg, vegetables, fruit, fish, and white meat) and 79 of which are classified as unhealthy (eg, chips, sugar-sweetened beverages, hot dogs, fried foods, and sugar-containing snacks). The classifications are based on a traffic light color coding of the nutrient content of the food developed by the Food Standards Agency (FSA) of the United Kingdom and the criteria for food and drinks described in the UK’s guide for nutritional labels [30]. These labels show at a glance if the food has low (green), medium (yellow), or high (red) amounts of fat, saturated fat, sugars, and salt, thus helping people achieve a better food balance. Each scenario replicates a real place where children commonly consume food in Mexico, including (1) a grocery store, (2) lunch café, (3) home-cooking restaurant, (4) fast food restaurant, (5) menu-based restaurant, and, as a bonus level, (6) a food truck. FoodRateMaster incorporates a curve of increasing difficulty across the 6 levels of the game to encourage players to have fun throughout to the end of the video game. The player is invited to play several times, to obtain three stars in each level, and to unlock access to an exclusive game zone. This zone presents an additional scenario (the 6th level) and a competitive challenge section where the winner of the challenge receives both their own points and those of an opponent.

#### FoodRateMaster Structure

**Figure 2:**
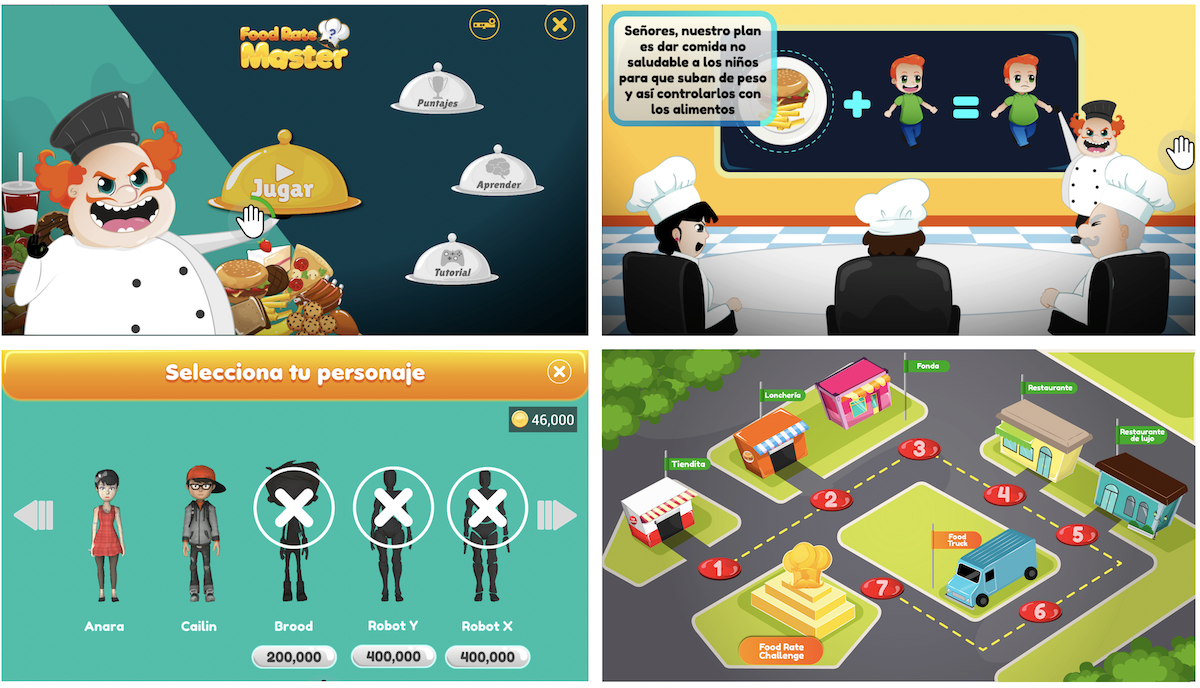
Screenshots of the FoodRateMaster conﬁguration/customization section. User menu (top left), game story (top right), avatar store and selection (bottom left), and scenario map (bottom right).

**Figure 3:**
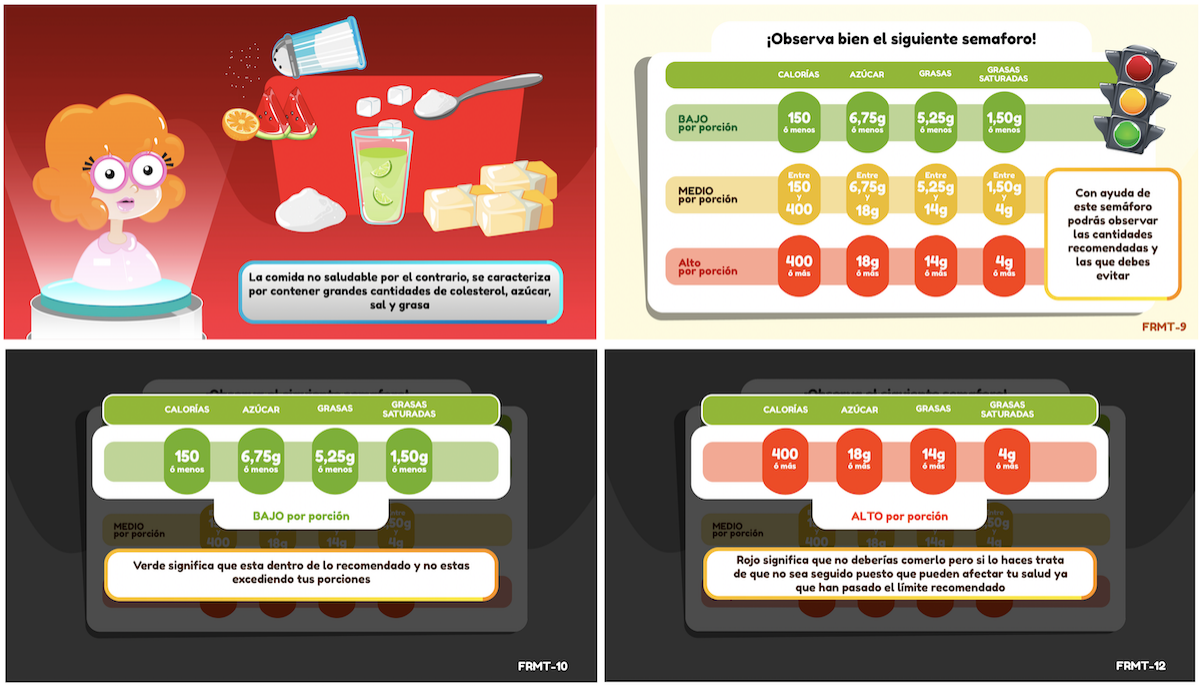
Screenshots of the FoodRateMaster educational section. Explanation of unhealthy food (top left), introduction to the traﬃc light color-coding system (top right), explanation of green levels (bottom left), explanation of red levels (bottom right).

**Figure 4:**
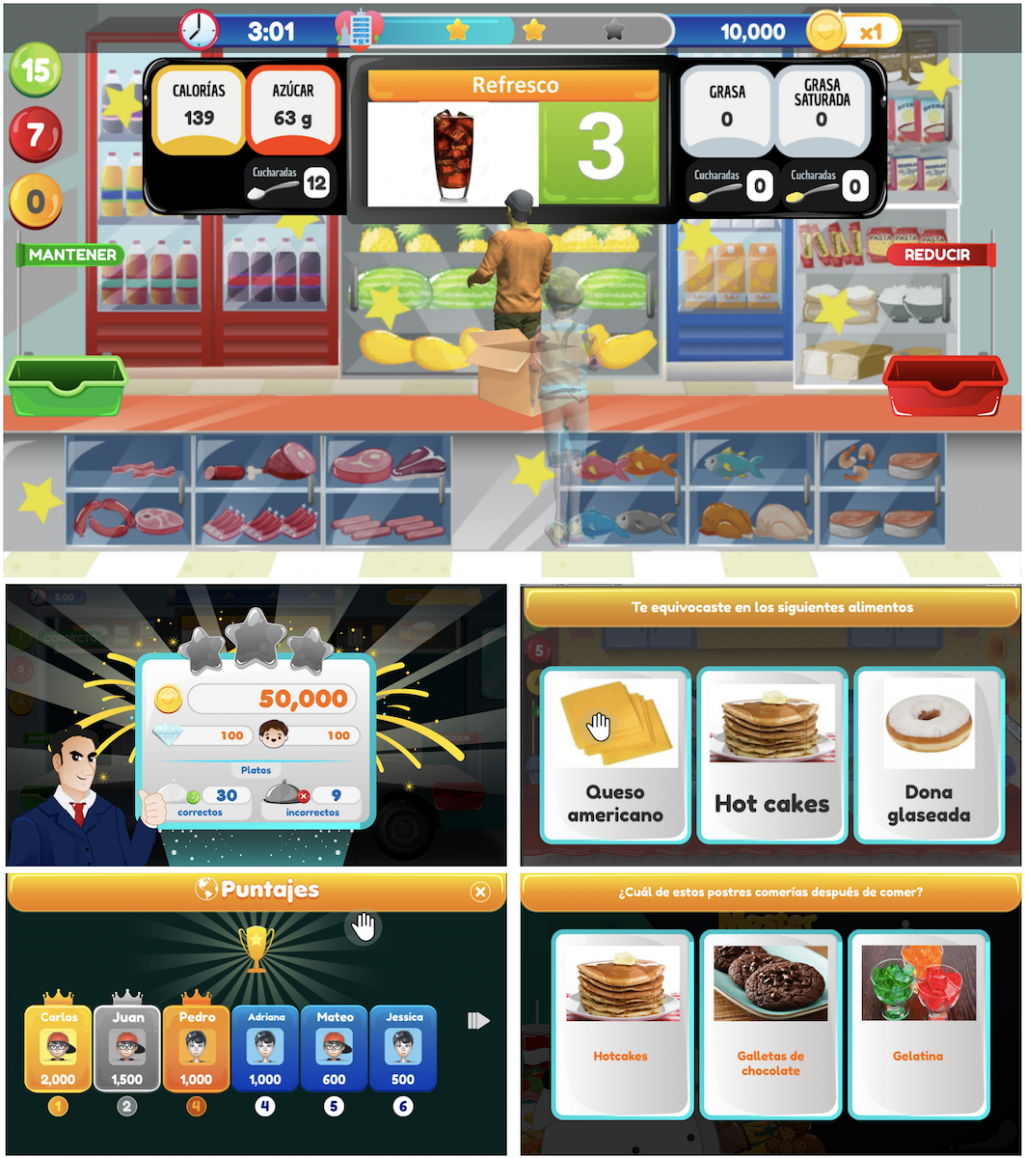
Screenshots of the FoodRateMaster training section. A game scenario (top), level results (middle left), error feedback (middle right), ranking of players (bottom left), and a validation question (bottom right).

FoodRateMaster has the following three sections: Section 1, “Configuration and customization” (see Figure 2); Section 2, “Education” (see Figure 3); and Section 3, “Training” (see Figure 4). Table 1 describes the activities for players in each section.

**Table 1:** Sections of FoodRateMaster.

Section	Description
Section 1: “Configuration”	Players log in, see the story of the game, select and buy avatars in the store, and select the scenario that they will play from the map.
Section 2: “Education”	Players learn about the characteristics of healthy and unhealthy food, and learn how to make healthier choices quickly and easily based on the traffic light color coding of the nutrient content of the food and drinks. Additionally, this tutorial explains the game goal, how to play the game, and what the game options, elements, indicators, and results section include.
Section 3: “Training”	In each scenario, an evil chef will prepare a menu, and the player, based on the nutritional information of the food, has to determine whether that food can be consumed (healthy) or whether its consumption must be reduced (unhealthy). The agent needs to take the healthy foods (only green labels) and put them into the “keep basket”. Additionally, the agent needs to take the unhealthy foods (yellow and red labels) and put them into the “reduce basket.” The player has to move his/her body to classify the food and avoid obstacles. During the game sessions, the game provides a bonus mechanism. The correct action of maintaining healthy food or reducing unhealthy food allows children to maintain a healthy lifestyle and therefore win points. Additionally, the player can earn extra points when he/she correctly replaces unhealthy food with healthy food. Otherwise, if the player makes too many errors, he/she will not win enough points to unlock the subsequent levels. Finally, the player arrives at the results screen, which specifies the points earned, the incorrect food classification choices he/she made, and his/her position in the global ranking of players. The player then answers some validation questions that help to identify what kind of food (healthy or unhealthy) they intend to consume in certain situations (eg, Which of the following foods would you eat after a meal?).

#### Behavioral Change Theories and Techniques

**Figure 5:**
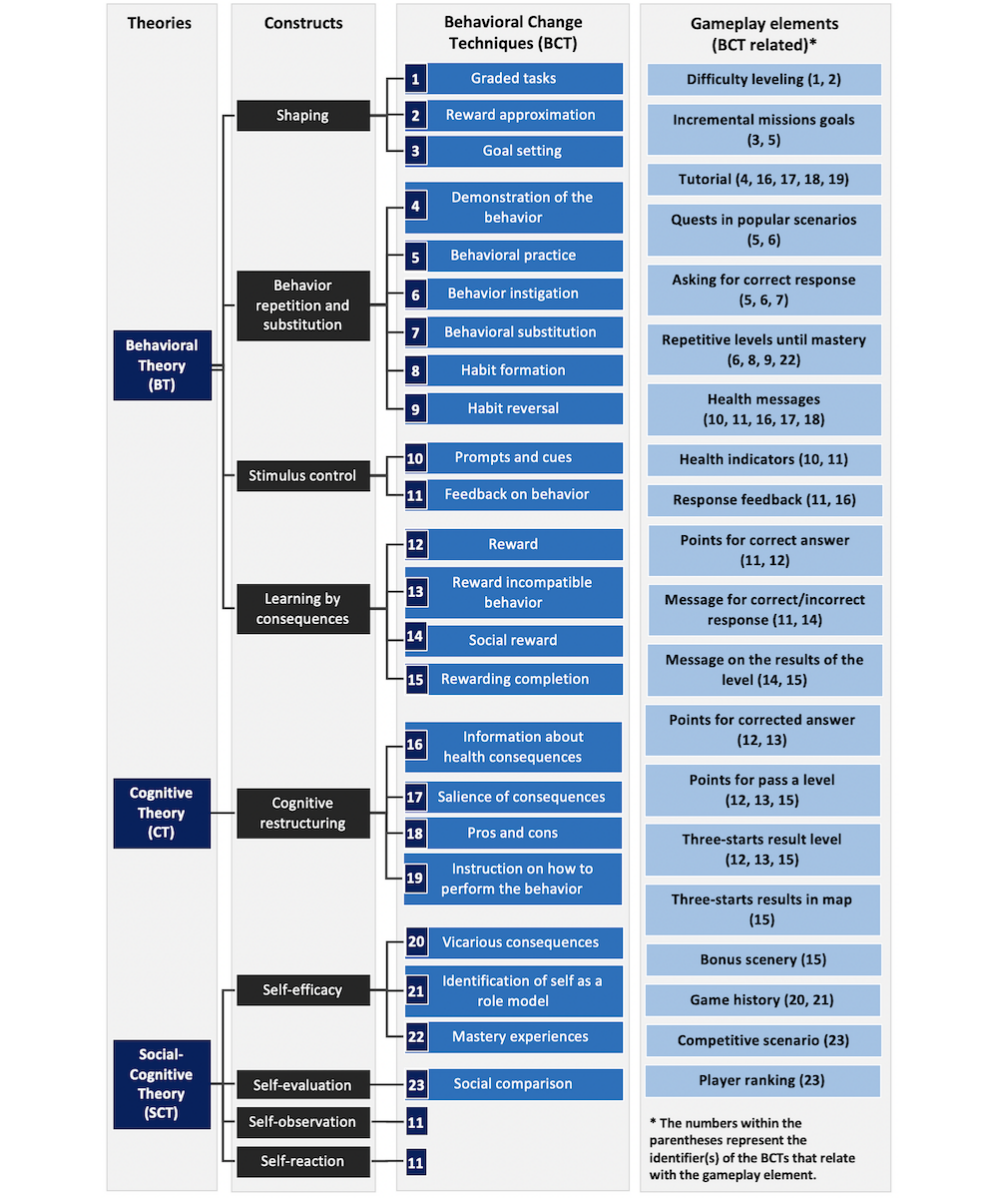
Theory-based gameplay elements in FoodRateMaster.

We included a set of BCTs in the gameplay elements of FoodRateMaster to create a stimulating and engaging environment in which key aspects of healthy behaviors and behavior-specific knowledge are being promoted and strengthened. A BCT is defined as “an observable, replicable, and irreducible component of an intervention designed to alter or redirect causal processes that regulate behavior” [31]. BCTs can be used alone, but the combination of several BCTs is frequently a keystone for effectiveness [32]. Figure 5 explicitly shows the relationship between the BCTs and gameplay elements. The BCTs included in FoodRateMaster are based on the work of Michie et al [31], and are grounded on the constructs of behavioral theory, cognitive theory, and social cognitive theory.

Behavioral theory suggests that the frequency, magnitude, and duration of a behavior (eg, exercising, consuming sugary drinks, or eating healthy) depends to a large extent on the consequences that the behavior produces or the elements of the context (eg, people, objects, and events) [33-35]. Thus, an essential feature of this approach focuses on the application of mechanisms or techniques that increase desirable healthy behavior and weaken or extinguish undesirable, troublesome, or unhealthy behaviors [36]. Behavioral therapy is primarily based on techniques such as shaping, stimulus control, behavior repetition and substitution, and learning by consequences [37,38].

Cognitive theory proposes that thoughts or cognitions play a definitive role in healthy behavior and in the general well-being of people [39,40]. This theory emphasizes how an individual processes, evaluates, and interprets reality, and how this information affects how the person behaves; the theory therefore focuses on reframing and correcting distorted thought patterns to facilitate behavioral change [41]. Cognitive therapy is based primarily on techniques such as cognitive restructuring, monitoring, and psychoeducation (ie, providing information about the problem).

Social cognitive theory emphasizes learning from the social environment and states that personal, behavioral, and environmental factors are interrelated and, in conjunction, influence behavior change [42]. Critical constructs of social cognitive theory are self-observation, self-evaluation, self-reaction, and self-efficacy.

Both behavioral and cognitive theories are supported by a large body of empirical evidence that supports their usefulness in the prevention and treatment of childhood obesity [43-45]. Similarly, social cognitive theory-based interventions have shown a positive effect on the prevention of childhood obesity [46].

### Participants

A total of 62 Mexican children (29 girls, 33 boys) aged 8-10 years (mean age 9, SD 0.8 years) attending a primary school located close to our research institution in Tepic, Nayarit, Mexico participated voluntarily in the study. The only exclusion criterion was having physical limitations, because the participant had to interact with the game through full-body movements. The 62 participants answered the initial questionnaires, but 2 of them did not answer the posttest questionnaires and were excluded from the study, resulting in 60 valid participants. The number of participants was 11, 35, and 14 for the age groups of 8, 9, and 10 years old, respectively. In addition, 39 parents of the participants answered the perception questionnaire.

### Procedures

We conducted a meeting with the school authorities and teachers to present FoodRateMaster, explain the objectives of the study, and verify that the children had not taken any nutrition education class that could influence the test. During this meeting, we obtained written authorization from the school authorities and teachers to conduct the study in their institution. Subsequently, a call for participants was administered by presenting FoodRateMaster to groups of children. In the presentation, we explained to the children the purpose of FoodRateMaster, its characteristics and elements, and the instructions about how to play the game. Written consent was obtained from the parents of the children who expressed their verbal interest in participating. The participants then completed the pretest questionnaire process as follows. During the first day, the facilitators grouped the participants by age (three groups) and asked them to complete the food knowledge questionnaire. The children took approximately 20 minutes to complete the questionnaire. Four days later, the children answered the food frequency questionnaire individually, which took approximately 30 minutes to complete. To improve the reliability of the data, children self-reported their food frequency intake. According to Kolodziejczyk et al [47], children’s self-reporting of dietary intake is more valid than parental reporting. In addition, the application was conducted with the close support of a facilitator. In this way, children only had to focus on remembering how often they had consumed the given food within the last month. Every child took the time they considered necessary to answer each question.

After filling out the questionnaires, the participants completed 12 game sessions of at least 15 minutes per session. The total average time of play was 3.5 hours. We conducted all game sessions in 45 days. In one room, we set up three game stations, each of which included a 50-inch monitor, personal computer, Kinect sensor V2, and the FoodRateMaster video game. We ensured that the participants always felt comfortable with the game activities. The day after the last game session, we began administering the posttest questionnaires, which followed the same procedure as the pretest questionnaires. Moreover, we asked the parents to complete the parent perception questionnaire. This questionnaire was anonymous, and the parents were instructed to return it within 1 week.

All study procedures were approved by the institutional review board of the Centro de Investigacion Cientifica y de Educacion Superior de Ensenada (Tepic, Nayarit, Mexico). In addition, we carefully ensured the anonymity, confidentiality, and safeguarding of data. Only data such as age, sex, and grade level were captured, and we did not collect the name or other sensitive data of the children.

### Measures

#### Food Knowledge Questionnaire

**Figure 6:**
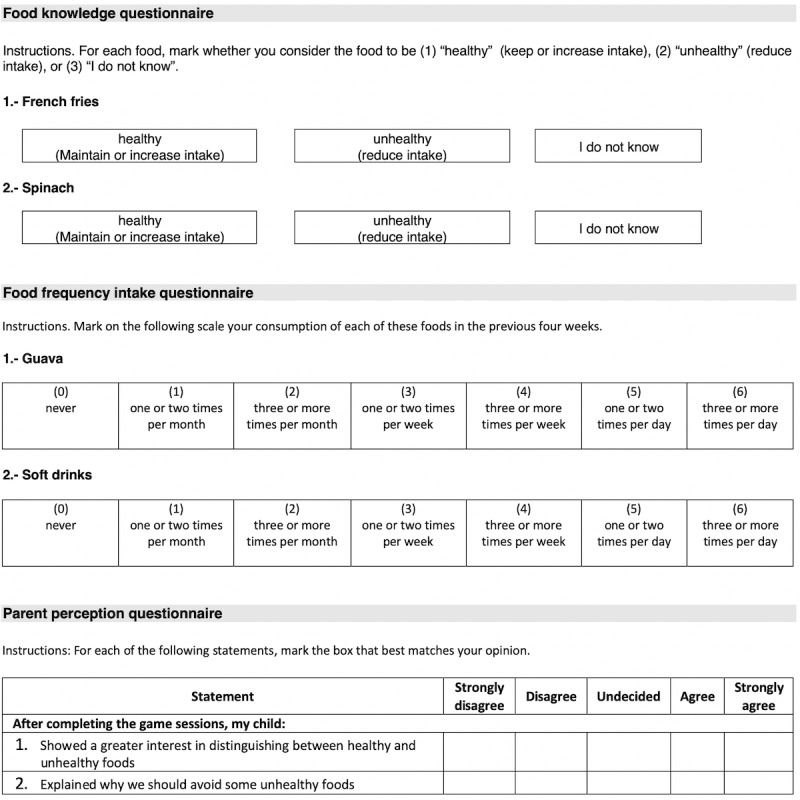
Example of questions from each questionnaire.

Figure 6 provides an example of the food knowledge questionnaire, which was developed by an interdisciplinary research team consisting of a nutritionist, a psychologist, and a computer scientist. The psychologist and the nutritionist also participated in some of the multidisciplinary design sessions. We developed this questionnaire because we did not find a validated questionnaire that evaluates this knowledge. This questionnaire included 90 foods, 49 of which were healthy and 41 of which were unhealthy. These foods were selected from interviews conducted with children before beginning this study. For each food, the participants were asked to indicate whether they considered the food to be (1) “healthy” (maintain or increase intake), (2) “unhealthy” (reduce intake), or (3) “I do not know.” The classification of each food as healthy or unhealthy was made based on the traffic light color coding of the nutrient content of the food developed by the UK FSA [30]. The total result for this questionnaire was the sum of the questions answered correctly. We carried out a pilot test of the questionnaire with 5 children to evaluate whether they had any problems answering it. We did not find any such incidents during this pilot test.

#### Food Frequency Questionnaire

As with the knowledge questionnaire, we did not find a valid food frequency questionnaire (FFQ) for school-age Mexican children. Therefore, a nutritionist and a computer scientist adapted the FFQ for children 7-10 years old developed by Hinnig et al [48] and the FFQ for assessing dietary intake in children and adolescents in South America developed by Saravia et al [49] to the diets of school-aged Mexican children (Figure 6). In addition, we considered the characteristics of the highest validated FFQ for children identified by Kolodziejczyk et al [47]. The adapted questionnaire includes 78 foods that were selected from interviews conducted with children prior to this study. Many of these foods are also included in the reference questionnaires. For each food, the participants were asked to mark on a 7-point scale the frequency of their consumption of the foods in the previous 4 weeks as follows: (0) “never,” (1) “one or two times per month,” (2) “three or four times per month,” (3) “one or two times per week,” (4) “three or more times per week,” (5) “one or two times per day,” and (6) “three or more times per day.” The items in this questionnaire were not interrelated and were analyzed individually. We also carried out a pilot test of this questionnaire with 5 children to evaluate whether they had any problems answering it. We did not encounter any such incidents during this pilot test.

#### Parent Perception Questionnaire

We developed the parent perception questionnaire to determine whether the participants’ parents perceived a positive change in their children’s attitudes toward 13 healthy eating behaviors after playing all of the game sessions. A nutritionist and a computer scientist developed this questionnaire. The first four questions were related to the following behaviors: distinguishing healthy and unhealthy foods, avoiding unhealthy foods, suggesting dietary changes for healthy eating, and reducing salt and sugar consumption. Of the remaining nine questions, four of them refer to behaviors associated with an increase in the intake of healthy foods (eg, consuming fruits and vegetables) and five refer to a reduction in the consumption of unhealthy foods (eg, reduce soft drink consumption). These healthy eating behaviors were selected because they are the most commonly recommended behaviors for healthy eating, and they are encouraged in FoodRateMaster. We asked the participants to indicate on a 5-point Likert-type scale ranging from (1) “totally disagree” (1) to “totally agree” (5) according to the level to which they believe that their child fits each statement. The items on this questionnaire were not interrelated and were analyzed individually. Additionally, this questionnaire included a final question asking the parents if they introduced any changes in the diets of their children due to the comments/suggestions from the children themselves. If the response to this question was “yes,” the parents were invited to write a free text explaining what changes they introduced.

### Data analysis

A Wilcoxon signed-rank sum test was used to determine whether the total number of correctly identified healthy and unhealthy foods differed from pre to post game play. We also conducted similar analyses for healthy and unhealthy food and the food categories. The food knowledge data (continuous variable) is presented as mean and SD. We used this nonparametric test because this variable did not exhibit a normal distribution. The Wilcoxon signed-rank sum test was also used to identify significant differences between the pretest and posttest results for the frequency of the consumption of the 78 foods included in the FFQ. We report the food frequency (categorical variable) as the median and interquartile range. We used this nonparametric test because this variable is ordinal. Finally, to determine whether the participants’ parents perceived changes in the attitudes of their children, we calculated the medians and interquartile range values, and the percentage of participant agreement as the average of the sums of percentages of participants who answered with option 4 “partially agree” or option 5 “totally agree” in each item of the subscale. We conducted the statistical analyses in the software SPSS version 25 (SPSS Inc, Chicago, IL, USA).

## Results

### Food Knowledge

The first hypothesis predicted that following game play, participants would be able to correctly identify a greater number of healthy and unhealthy foods. Indeed, participants increased the number of foods they correctly identified from pregame tests. We also identified significant differences in the food classification of both healthy and unhealthy foods, but the players’ improvement in correctly identified foods was more significant with healthy than with unhealthy food. Overall, we identified significant differences in the knowledge of players in almost all food categories except for the category of sugar with fat. Table 2 summarizes the complete results.

**Table 2:** Pretest and posttest results of the food knowledge questionnaire.

Food group		Questions, N	Pretest, mean (SD)	Posttest, mean (SD)	*P* value^a^	Change
All foods		90	56.95 (10.71)	67.88 (10.71)	<.001	+19%
**Healthy and unhealthy foods**						
	Healthy foods	49	29.13 (6.47)	35.72 (7.03)	<.001	+23%
	Unhealthy foods	41	27.67 (9.01)	32.37 (6.43)	<.001	+16%
**Food category**						
	Animal-derived food	5	1.70 (1.28)	2.57 (1.24)	<.001	+47%
	Cereals with fat	16	10.83 (3.46)	13.00 (2.54)	<.001	+19%
	Fast food	3	2.10 (1.07)	2.38 (0.83)	.002	+14%
	Fat-free cereals	8	4.27 (1.34)	5.25 (1.34)	<.001	+23%
	Fat-free sugar	10	7.38 (1.65)	8.58 (1.65)	<.001	+16%
	Fruit	7	6.07 (1.02)	6.58 (0.94)	<.001	+9%
	Legumes	3	1.73 (0.63)	2.03 (0.78)	<.001	+18%
	Oils and fats	6	3.97 (1.34)	5.00 (0.99)	<.001	+27%
	Prepared food	23	11.68 (3.42)	14.98 (3.70)	<.001	+24%
	Sugar with fat	3	2.15 (0.90)	2.30 (0.72)	.22	—^b^
	Vegetables	6	4.92 (1.29)	5.40 (0.98)	<.001	+11%

^a^*P* value of <.05 was considered statistically significant.

^b^Not applicable.

### Food Frequency Intake

Hypothesis 2 surmised that participants would exhibit an increased self-reported food frequency intake of healthy food. Indeed, the participants indicated a greater self-reported food frequency intake of cauliflower, broccoli, and corn quesadillas. Therefore, hypothesis 2 was confirmed. In addition, hypothesis 3 surmised that participants would exhibit a reduced self-reported food frequency intake of unhealthy food. After game play, participants indicated a reduced self-reported frequency intake of french fries, pancakes, brownies and donuts, candy and chocolate, crackers, wheat tortillas, crepes, sweet soft cakes, sweet cookies, hamburgers, and soft drinks. These results also confirm hypothesis 3. Table 3 summarizes the complete results on food frequency intake.

**Table 3:** ﻿ Pretest and posttest results of the food frequency questionnaire.

Food		Pretest score^a^, median (IQR)	Posttest score, median (IQR)	*P* value^b^	Change
**Healthy food**					
	Cauliflower, broccoli	0 (0-3)	3 (1-4)	<.001	increase
	Corn quesadillas	0 (0-3)	3 (1-3)	<.001	increase
	Corncob	3 (1.25-5)	3 (1-3)	<.001	decrease
	Guava	1.5 (0-4.75)	0 (0-2)	<.001	decrease
**Unhealthy food**					
	French fries	1 (0-3)	1 (0-1)	.003	decrease
	Crackers	3 (0-3)	1 (0-1.75)	.004	decrease
	Wheat tortillas	3 (0.25-5)	2 (0-3.75)	.03	decrease
	Pancakes, brownies, donuts	3 (1-5)	1 (0-3)	<.001	decrease
	Crepes	1 (0-3)	1 (0-1)	.01	decrease
	Candy, chocolate	4 (1.25-5)	1.5 (0.25-2.75)	<.001	decrease
	Sweet soft cakes	1 (0-3)	0 (0-1)	.009	decrease
	Sweet cookies	3.5 (3-5)	3 (2.25-4.75)	.02	decrease
	Soft drinks	3 (0-4.75)	1 (0-3)	.03	decrease
	Hamburgers	1 (0-3)	1 (0-2)	.02	decrease

^a^The scores are (0) “never,” (1) “one or two times per month,” (2) “three or more times per month,” (3) “one or two times per week,” (4) “three or more times per week,” (5) “one or two times per day,” and (6) “three or more times per day.”

^b^*P* value of <.05 was considered statistically significant.

### Parent Perceptions

Finally, hypothesis 4 surmised that the participants’ parents would perceive improvements in their children’s attitudes toward healthy eating behaviors. Most of the participants’ parents (>50%) agreed that they perceived improvement in the attitudes of their children toward 6 of the 13 healthy eating behaviors after playing the game (see Table 4). In particular, most of the parents agreed that after playing the game, their children showed a greater interest in explaining why they should avoid unhealthy foods, distinguish between healthy and unhealthy foods, increase their intake of fruits and vegetables, reduce their intake of soft drinks, and suggest changes in the intake of some foods that they usually eat. The remaining behaviors did not reach 50% parental agreement. Additionally, a total of 15 parents answered the final question related to the introduction of any change in the regular diet of their children due to the comments/suggestions the children made (see Textbox 1). Nine comments were related to a reduction of unhealthy food intake, five were related to an increase in healthy food intake, and one was related to the learning of content but not the intention of changing eating behaviors.

**Table 4:** Parent perception questionnaire responses (N=39).

After completing the game sessions, my child:	Score^a^, median (IQR)	Agree^b^, n (%)
Showed greater interest in distinguishing between healthy and unhealthy foods	4 (2-5)	25 (64)
Explained why we should avoid some unhealthy foods	4 (3-5)	26 (67)
Suggested changing the consumption of some foods that we usually eat	4 (1.5-4)	20 (51)
Showed greater interest in the reduction of salt and sugar within foods	3 (2-5)	19 (49)
Showed greater interest in the consumption of legumes	3 (2-5)	19 (49)
Showed greater interest in the consumption of seafood	3 (2-5)	19 (49)
Showed greater interest in the consumption of vegetables	4 (2.5-5)	23 (59)
Showed greater interest in the consumption of fruits	4 (2-5)	25 (64)
Showed greater interest in reducing the consumption of fried foods	3 (2-4)	16 (41)
Showed greater interest in reducing the consumption of soft drinks	4 (2.5-5)	21 (54)
Showed greater interest in reducing the consumption of fast food	3 (2-5)	19 (49)
Showed greater interest in reducing the consumption of candy and chocolate	3 (2-4)	17 (44)
Showed greater interest in reducing the consumption of cookies and pancakes	3 (2-4)	18 (46)

^a^Scores are based on a choice of the following options: 1, strongly disagree; 2, disagree; 3, undecided; 4, agree; 5, strongly agree.

^b^Responded 4 (agree) or 5 (strongly agree).

**Collected comments from the parent perception questionnaire.**

- Related to the consumption of healthy food

“I noticed that he more easily accepts the fruits and vegetables that he has to eat.”

“My child now better accepts the consumption of vegetables. I noted that he is now more sensitive to obesity, and I heard him say that he should exercise more.”

“My child now eats vegetables and meat better.”

“Yes, now he is getting used to eating more fruits and vegetables.”

“Now we try to have a healthier diet all the time and inform our children about the properties of food.”

- Related to the consumption of unhealthy food

“I am now trying to reduce the use of sugar, salt, and fat when cooking.”

“We have reduced the consumption of sodas, candies, sugar-sweetened bread, and snacks.”

“We now avoid the consumption of hamburgers, hot dogs, and soft drinks.”

“My son no longer shows so much interest in or taste for candies or chocolates.”

“Now my child drinks less soda.”

“Now my child eats fewer french fries and chips.”

“We have reduced the consumption of salt and fast food.”

“I think that every child is attracted to junk food, but now [he/she] only occasionally consumes junk food.”

“Now my child drinks less soda and sugar-sweetened beverages than before.”

- Related to learning but not to the intention of changing food behaviors

“He told me about the game, and it is about what does and what does not benefit his body, but he does not have an interest in changing his eating habits. Every day is a constant struggle to try different fruits and vegetables.”

## Discussion

### Principal Findings

The findings from this study support the ability of FoodRateMaster to help improve the nutritional knowledge and dietary intake of children between 8 and 10 years old, which corresponds to the age range at which the rates of overweight or obesity in children increase considerably [3]. In addition, parents perceived positive changes in their children’s attitudes toward healthy eating behaviors after game play.

### Food Knowledge

The results obtained in this study show that children aged between 8 and 10 years old significantly improved their food knowledge after playing FoodRateMaster. This result supports prior evidence about the usefulness of serious games to improve food nutrition knowledge (eg, [11,15]). In particular, we identified a greater increase in the ability to correctly identify healthy food than in the ability to correctly identify unhealthy food. This demonstrates that it is challenging to change the opinion of children about unhealthy food that is wrongly perceived as healthy. Previous studies suggest that this situation might occur because children heavily rely on visual aspects to assess the healthiness of a food product and because they have difficulty classifying the combined and transformed food products with which they are most likely to be presented in their everyday lives [50]. Transformed foods are defined as foods in which a sign of human intervention can be traced (eg, mayonnaise, sweet cookies, and hamburgers) [51]. Additionally, our findings suggest that knowledge improvement varies between different food categories. The categories that showed the least improvement were fruits/vegetables and fast food. We attribute these results to the fact that these foods are frequently publicized as healthy and unhealthy food, respectively. The categories with the most significant improvement were animal-derived food, oils and fats, prepared food, and cereals without fat. These findings suggest that future initiatives should concentrate on the greater exposition of food categories, which can contribute to correct identification.

### Food Frequency Intake

We also obtained positive results when assessing the changes in the food frequency intake of healthy and unhealthy foods. A statistically significant change was found in the frequency of consumption of 14 of 78 foods. Most of these changes (10 of 14) consisted of the reduced consumption of unhealthy foods such as soft drinks, pancakes, donuts, brownies, and candy, especially since these foods are usually consumed in both the schools and homes of Mexican children [8]. These results support the usefulness of serious games in improving dietary intake and are consistent with previous studies (eg, [15,18]). Complementarily, only two changes were associated with the increasing consumption of healthy food (eg, cauliflower and broccoli). Previous studies suggest that this situation occurs because providing children with information or visual exposure to foods alone may not be a sufficient mechanism for increasing children’s preference and consumption of healthy foods [52,53]. A strategy that is more effective for increasing children’s preference of healthy foods is repeated exposure to these foods [52]. These findings suggest that future initiatives should include other BCTs that could be effective in improving children’s intake of fruits, vegetables, and other healthy foods (eg, self-monitoring, social support, goal setting, goal review, and action planning). It is also important to note the decrease in the consumption of guava (a local fruit) and corn on the cob, both of which are foods that are classified as healthy. This reduction might be caused by the coincidence of the end of the harvesting season of these foods during the study period.

### Parent Perceptions

The feedback collected from the participants’ parents provides a complementary source of information, as the parents are responsible for the type of foods the children usually consume. Therefore, it is important to determine whether the parents noted any change in the attitudes of their children toward the intake of healthy and unhealthy food. The most relevant feedback collected from the parents (ie, higher percentages of agreement presented in Table 4) indicates that most of the parents agreed that after playing the game, their children showed greater interest in the following six healthy eating behaviors: explaining why they should avoid some unhealthy foods, distinguishing between healthy and unhealthy foods, increasing their intake of fruits and vegetables, reducing their intake of soft drinks, and suggesting changes in the intake of some foods that they usually eat. These results support the argument that FoodRateMaster can influence children’s attitudes toward healthy eating behaviors. Conversely, the percentage of parents who agreed that they perceived changes in their children’s attitudes did not exceed 50% for the remaining seven healthy behaviors. According to Ledoux et al [54], one explanation for this result is that the expectations parents have of their children influence the way they interpret their children’s behaviors, and many expectations that parents have of their children may be unrealistic. In addition, 14 parents commented that they introduced some changes in their children’s diet based on the comments and suggestions they received from their children (eg, “Yes, now he is getting used to eating more fruits and vegetables” or “Now my child drinks less soda”). These results support the argument that FoodRateMaster can influence children to involve their parents in changing their healthy and unhealthy eating behaviors. Interventions in which parents are actively involved in the improvement of their child’s dietary intake are more likely to result in positive outcomes [55].

### Limitations and Future Work

The project was evaluated in an uncontrolled clinical trial involving a small number of participants. Therefore, the results should be interpreted with caution. However, given that our main objective was to carry out a pilot study to explore the feasibility of FoodRateMaster to support nutritional learning and changes in food eating behaviors of children aged between 8 and 10 years, we believe that our results are valuable for researchers exploring the design of this type of serious game for players in that age range. A controlled clinical trial with a much larger population is necessary to prove the effectiveness of FoodRateMaster and is part of planned future work.

Another limitation of this study was the short evaluation period. The children answered the posttest questionnaires 4 days after the game sessions were complete. Medium-term and long-term effects of FoodRateMaster were not addressed herein and could constitute insights for future research. One possibility would be to increase the time lag between exposure and measurement to account for medium-term effects. Another suggestion would be to evaluate the long-term effects by using a longitudinal design with repeated exposure effects. In addition, it would be interesting to expand the population involved in this pilot study and conduct a long-term study to analyze the effect of FoodRateMaster in children who are overweight or obese.

In addition, the food frequency intake was based on self-reported data. The results may be biased by well-known limitations of FFQs, such as the difficulty in remembering experiences or events and the exaggeration or underreporting of food intake [56]. However, an FFQ is the most commonly used method for such assessments owing to its ease of use and its reliability and validity in capturing such information. There is also evidence that the self-reporting of dietary intake by children is more valid than parental reporting [47]. In addition, the present study did not examine changes in the number of servings consumed by children. Instead, only changes in food frequency intake were considered. Therefore, it would be interesting to address this nutritional assessment comparison before and after game exposure to better evaluate positive or negative changes in food servings. However, the most accurate instruments to measure food intake in children are those that do not include the number of servings [47].

Moreover, we only obtained data on the extent to which parents perceived any change in the attitudes of their children toward healthy eating behaviors, and we did not capture information that could help to explain this perception. In a future evaluation, we will include questions that will help us to identify the specific aspects that influence this perception.

### Conclusions

In this study, extensive formative research was conducted by a multidisciplinary team to design and develop FoodRateMaster, a health video game for nutritional education and the promotion of healthy eating behaviors among young children. Compared to the initial evaluation, children aged between 8 and 10 years indicated an increased level of nutritional knowledge and self-reported frequency intake of two healthy foods, and a decreased level of self-reported intake of 10 unhealthy foods after playing FoodRateMaster. Moreover, the participants’ parents agreed that FoodRateMaster positively influenced their children’s attitudes toward several healthy eating behaviors. These results support that a health game such as FoodRateMaster is a viable tool to help children aged between 8 and 10 years old to increase their food knowledge and improve their dietary behaviors.

The results from this study also suggest that greater exposure to some food categories may be needed to increase children’s knowledge about healthy and unhealthy foods. In addition, future versions of FoodRateMaster should include other BCTs that can be useful in improving children’s intake of fruits, vegetables, and others healthy foods. For future research, we are planning to conduct a randomized controlled trial to evaluate the medium-term and long-term effects of FoodRateMaster.

## Abbreviations

BCT: behavior change technique
FFQ: food frequency questionnaire
FSA: Food Standards Agency of the United Kingdom
